# An Improved Reduced-Dimension Robust Capon Beamforming Method Using Krylov Subspace Techniques

**DOI:** 10.3390/s24227152

**Published:** 2024-11-07

**Authors:** Xiaolin Wang, Xihai Jiang, Yaowu Chen

**Affiliations:** 1 College of Biomedical Engineering and Instrument Science, Zhejiang University, Hangzhou 310027, China; cyw@mail.bme.zju.edu.cn; 2 Hangzhou Applied Acoustics Research Institute, Hangzhou 311400, China; jxh_book@163.com

**Keywords:** robust adaptive beamforming, Krylov subspace methods, Newton search, computational cost

## Abstract

A reduced-dimension robust Capon beamforming method using Krylov subspace techniques (RDRCB) is a diagonal loading algorithm with low complexity, fast convergence and strong anti-interference ability. The diagonal loading level of RDRCB is known to become invalid if the initial value of the Newton iteration method is incorrect and the Hessel matrix is non-positive definite. To improve the robustness of RDRCB, an improved RDRCB (IRDRCB) was proposed in this study. We analyzed the variation in the loading factor with the eigenvalues of the reduced-dimensional covariance matrix and derived the upper and lower boundaries of the diagonal loading level; the diagonal loading level of the IRDRCB was kept within the bounds mentioned above. The computer simulation results show that the IRDRCB can effectively solve the problems of a sharp decline in the signal-to-noise ratio gain and an invalid diagonal loading level. The experimental results demonstrate that the interference noise of the IRDRCB is 3~5 dB higher than that of conventional adaptive beamforming, and the computational complexity is reduced by 15% to 20% compared with that of the RCB method.

## 1. Introduction

Beamforming is often used in passive sonar applications, such as extracting spatial information about impinging signals, source localization, separating mixtures of impinging signals [1,2], improving the detectability of weak sources [3,4] and more. Because the conventional beamformer is only optimal for the estimation of the spatial parameters of sources that are modeled as a single source embedded in spatially white noise, the trend has been to employ adaptive beamforming techniques.

A minimum variance distortionless response (MVDR) can effectively suppress interference and noise by adaptively producing null in the direction of interference (DOI), which is also known as the Capon beamformer [5,6]. Although the Capon has a better resolution and much better interference rejection capability, it is not widely used in passive sonar due to its lack of robustness in the presence of array steering vector errors. A finite number of snapshots, model mismatch and sample errors are the main sources of array steering vector (ASV) errors; in particular, when the covariance matrix contains the desired signal, the Capon may mistakenly suppress the signal of interest (SOI) as if it were interference, significantly reducing the estimated signal power [7,8]. The phenomenon of underestimating SOI power is called “SOI cancellation”. Many approaches to increasing the robustness and reliability of the Capon in engineering applications have emerged. Among these, the diagonal loading method has been used most widely to alleviate the deleterious effects of ASV mismatch [9,10], but its disadvantage is that the same diagonal loading level is chosen for all the frequency covariance matrices, which makes it easy for some frequency components with good detection performance to be overloaded, affecting broadband detection performance. Robust Capon beamforming (RCB) based on an uncertainty set of the array steering vector with a clear theoretical background has been proposed, for which the corresponding amount of diagonal loading can be calculated precisely according to steering vector error [11,12]. In [13], a norm constraint on the weight vector is used to improve the robustness of RCB against array steering vector error and noise. In [14], a new approach is proposed based on the optimization of the worst-case performance, which minimizes the quadratic function subject to infinitely many nonconvex quadratic constraints. The doubly constrained robust Capon beamformer exploits a constant norm constraint and a spherical uncertainty set constraint to address steering vector error [15]. When large steering vector mismatches occur, an iterative robust minimum variance beamformer is required to achieve robustness against large steering direction errors by iteratively searching for the desired array steering vector [16].

With the need for long-range target detection, sonar systems are evolving towards having large apertures and many-element arrays, but the complexity $\vartheta (M^{3})$ of the eigenvalue decomposition in solving the covariance matrix is prohibitive to real-time implementation on large arrays. Many reduced-dimension methods for RCB have been proposed, including an iterative algorithm [17,18], a beam-space algorithm [19,20] and other reduced-dimension methods [21,22]. A Krylov subspace is a typical reduced-rank method widely used in passive sonar; RDRCB accelerates the convergence of RCB by exploiting Krylov subspace methods [23,24]. These Krylov subspace methods include the conjugate gradient (CG) technique [25,26], the multistage Wiener filter [27,28] and the auxiliary vector method [29,30]. Computer simulation results show that CG techniques require significantly fewer operation counts than other reduced-rank techniques.

Reduced-rank linear filtering and the calculation of the diagonal loading level are the most important processes of RDRCB methods. Reduced-rank linear filtering achieves near full-rank performance and enables accurate power estimation with a relatively small number of operation counts. Two existing stopping criteria proposed in [31] can achieve near full-rank performance with a filter rank much lower than the number of sensors. However, the diagonal loading level of RDRCB is calculated precisely with the Newton iteration method in [23]; if the initial value determined using the Newton iteration method is incorrect and the Hessel matrix is non-positive definite, the diagonal loading level of RDRCB becomes invalid. To obtain a valid diagonal loading level, an improved RDRCB was proposed in this study. Additionally, we provided insights into the variation in the loading level with the eigenvalues of the reduced-dimensional covariance matrix and derived the upper and lower boundaries of the diagonal loading level. Once the loading level was outside the boundaries, the solution process was terminated by the stopping criteria and recalculated using the bisection method. The computer simulation results show that the IRDRCB can effectively solve the problems of a sharp decline in the signal-to-noise ratio gain and an invalid loading level. The experimental results demonstrate that the interference noise of the IRDRCB was 3~5 dB higher than that of conventional adaptive beamforming, while the computational complexity was reduced by 15% to 20% compared with that of RCB.

The rest of the paper is organized as follows: Section 2 provides insights into the variation in the loading level with the eigenvalues of the reduced-dimensional covariance matrix, and describes the derivation of the upper and lower boundaries of the diagonal loading level for the IRDRCB methods. Section 3 presents numerical examples and experimental data, comparing the performance of RCB and the MVDR. Finally, conclusions are drawn in Section 4.

## 2. An Improved Reduced-Dimension Robust Capon Beamforming Method Using Krylov Subspace Techniques

### 2.1. Conjugate Gradient Method

Using the approach outlined in [23], the N-dimensional dimension-reducing transform $D=\left[d_{1}\cdots d_{N}\right]$ can be obtained using the conjugate gradient algorithm (CG). The main steps for computing the reduced-dimension ellipsoids are as follows.

Let r and d be the residual vector and the direction vector for the CG method applied to RCB.

The initial conditions are set as follows: $d_{1}=\bar{a},r_{1}=-\bar{a},i=1,\cdots ,N-1$.

The step size, $\alpha_{i}$, is calculated:

(1) $$\alpha_{i}=-\frac{d_{i}r_{i}}{d_{i}^{H}{\hat{R}}_{x}d_{i}}$$

where ${\hat{R}}_{x}$ is the M×M sample covariance matrix of the input array data vector $x\in C^{M}$, $\alpha_{i}$ is the step size that minimizes the cost function, $d_{i}$ is the direction vector, and the residual vector is given by

(2) $$r_{i+1}=r_{i}+\alpha_{i}{\hat{R}}_{x}d_{i}$$

$\beta_{i}$ provides orthogonality for the direction vector $d_{i}$ and is defined as

(3) $$\beta_{i}=\frac{d_{i}^{H}{\hat{R}}_{x}r_{i}}{d_{i}^{H}{\hat{R}}_{x}d_{i}}$$

The direction vector $d_{i+1}$ is then computed as

(4) $$d_{i+1}=-r_{i+1}+\beta_{i}d_{i}$$

### 2.2. Fast Conjugate Gradient-Based RDRCB

Here, we first summarize the existing RDRCB framework, proposed in [23,24], for combining Krylov subspace dimension reduction with RCB.

The kth array snapshot $x_{k}\in C^{M}$ is described as

(5) $$x_{k}=a_{0}s_{k}+\sum_{i=1}^{P}a_{i}r_{i}+n_{k}$$

where s, r and n are the desired signal, interference and noise, assuming the desired signal and interferers are uncorrelated and stationary. Here, the signal steering vector is $a_{0}$, and P is the number of interference targets; the array covariance matrix $R_{x}$ is modeled as

(6) $$R_{x}=E\left\{x_{k}x_{k}^{H}\right\}=\overset{2}{\underset{0}{\sigma }}a_{0}a_{0}^{H}+\sum_{i=1}^{P}\sigma_{i}^{2}a_{i}a_{i}^{H}+Q_{x}$$

where $\sigma_{0}^{2}$ and $\sigma_{i}^{2}$ are the power of the signal and the interference, and $Q_{x}=E\left\{n_{k}n_{k}^{H}\right\}$ is the M×M noise covariance matrix. In practice, $R_{x}$ is commonly replaced with the sample covariance matrix estimate:

(7) $${\hat{R}}_{x}=\frac{1}{K}\sum_{k=1}^{K}x_{k}x_{k}^{H}$$

The key is noting that an orthogonal basis $D=\left[d_{1}\cdots d_{N}\right]$ is formed using the CG algorithm:

(8) $${\hat{R}}_{y}=D^{H}{\hat{R}}_{x}D=\Lambda_{CG}$$

where $\Lambda_{CG}$ is a diagonal matrix and can be written as

(9) $$\Lambda_{CG}=diag\{[d_{1}^{H}{\hat{R}}_{x}d_{1},\cdots ,d_{N}^{H}{\hat{R}}_{x}d_{N}]\}$$

In general, the RDRCB framework is designed according to

(10) $$\min b^{H}R_{y}^{-1}b,s.t.[b-\bar{b}{]}^{H}F[b-\bar{b}]\leq 1$$

where $b=D^{H}a$ is the true reduced-rank steering vector of the SOI, $\bar{b}=D^{H}\bar{a}$ is the assumed reduced-rank steering vector of the SOI, and $F=\varepsilon^{-1}(D^{H}D{)}^{-1}$, ε is a user parameter. Note that $F\geq 0\in C^{N\times N}$ (9) can be rewritten as

(11) $$\min b^{H}\Lambda_{CG}^{-1}b,s.t.[b-\bar{b}{]}^{H}F[b-\bar{b}]\leq 1$$

Letting $M=\Lambda_{CG}^{\frac{-1}{2}}D^{H}ED\Lambda_{CG}^{\frac{-H}{2}}$, $\overset{˘}{b}=\Lambda_{CG}^{\frac{-1}{2}}b$, and $\overset{˘}{\bar{b}}=\Lambda_{CG}^{\frac{-1}{2}}\bar{b}$, the RDRCB framework can be reduced to

(12) $$min{\overset{˘}{b}}^{H}\overset{˘}{b},s.t.[\overset{˘}{b}-\overset{˘}{\bar{b}}{]}^{H}M^{-1}[\overset{˘}{b}-\overset{˘}{\bar{b}}]\leq 1$$

Using the approach outlined in [22], the associated Lagrangian function is formed as

(13) $$L\left(\overset{˘}{b},\mu \right)={\overset{˘}{b}}^{H}\overset{˘}{b}+\mu \left([\overset{˘}{b}-\overset{˘}{\bar{b}}{]}^{H}M^{-1}[\overset{˘}{b}-\overset{˘}{\bar{b}}]-1\right)$$

where μ is a Lagrange multiplier. Letting $\frac{\partial L\left(\overset{˘}{b},\mu \right)}{\partial {\overset{˘}{b}}^{H}}=0$ yields

(14) $$\hat{\overset{˘}{b}}={\left(\frac{M}{\mu }+I\right)}^{-1}\overset{˘}{\bar{b}}=\overset{˘}{\bar{b}}-{\left[\mu M^{-1}+I\right]}^{-1}\overset{˘}{\bar{b}}$$

Note that by using (14) in (13), we can rewrite (13) as

(15) $$h(\overset{˘}{\bar{b}},\mu )={\overset{˘}{\bar{b}}}^{H}{\left[\mu M^{-1}+I\right]}^{-1}M^{-1}{\left[\mu M^{-1}+I\right]}^{-1}\overset{˘}{\bar{b}}$$

We compute the eigendecomposition of M

(16) $$M=U\Lambda U^{H}$$

where the columns of U contain the eigenvectors of M and $\lambda_{1}\geq \lambda_{2}\geq \cdots \geq \lambda_{N}$ is the corresponding eigenvalue; let

(17) $$c=U^{H}\overset{˘}{\bar{b}}$$

where $c_{n}$ denotes the nth element of c, and (17) can be written as

(18) $$h(\overset{˘}{\bar{b}},\mu )=\sum_{n=1}^{N}\frac{\lambda_{n}{\left|c_{n}\right|}^{2}}{{\left(\mu +\lambda_{n}\right)}^{2}}$$

$h(\overset{˘}{\bar{b}},\mu )$ is a monotonically decreasing function of μ>0; using μ=0, we obtain

(19) $$h(\overset{˘}{\bar{b}},\mu )={\overset{˘}{\bar{b}}}^{H}M^{-1}\overset{˘}{\bar{b}}={\bar{b}}^{H}F\bar{b}$$

Thus, to avoid obtaining an invalid solution, we require that ${\bar{b}}^{H}F\bar{b}>1$; if satisfied, it means that μ≠0. The Lagrange multiplier can be found using the Newton search. Once μ has been found, $\hat{\overset{˘}{b}}$ is found using (14), and the weights are then formed as

(20) $$W_{RDRCB}=\frac{\Lambda_{CG}^{-1}{\hat{b}}_{0}}{{\hat{b}}_{0}^{H}\Lambda_{CG}^{-1}{\hat{b}}_{0}}$$

where ${\hat{b}}_{0}=\Lambda_{CG}^{\frac{1}{2}}\hat{\overset{˘}{b}}$.

The main steps for computing the RDRCB weights are as follows.

Step (1) Calculate the covariance matrix ${\hat{R}}_{x}$.

Step (2) Evaluate the DRT D (using the CG transform) and the diagonal matrix.

Step (3) Form $\overset{˘}{\bar{b}}=\Lambda_{CG}^{-\frac{1}{2}}D^{H}\bar{a}$ and $M=\Lambda_{CG}^{-\frac{1}{2}}D^{H}E^{-1}D\Lambda_{CG}^{-\frac{H}{2}}$.

Step (4) Form $c=U^{H}\overset{˘}{\bar{b}}$ and find the solution μ>0 to $h(\overset{˘}{\bar{b}},\mu )=1$ via the Newton search.

Step (5) Form $\hat{\overset{˘}{b}}=\overset{˘}{\bar{b}}-U{\left[\mu_{opt}\Lambda^{-1}+I\right]}^{-1}c$.

Step (6) Form ${\hat{b}}_{0}=\Lambda_{CG}^{\frac{1}{2}}\hat{\overset{˘}{b}}$ and $W_{RDRCB}=\frac{\Lambda_{CG}^{-1}{\hat{b}}_{0}}{{\hat{b}}_{0}^{H}\Lambda_{CG}^{-1}{\hat{b}}_{0}}$.

### 2.3. The Upper and Lower Boundaries of the Lagrange Multiplier

The Newton method is an iterative algorithm with the advantage of fast convergence, which ensures sufficiently low complexity to permit real-time implementation in passive sonar signal processing, so the diagonal loading level of RDRCB is calculated precisely through the Newton search. However, if the initial value determined with the Newton iteration method is incorrect and the Hessel matrix is non-positive definite, the diagonal loading level of RDRCB becomes invalid. To obtain a correct diagonal loading level, it is imperative to improve the robustness of RDRCB using the Newton search. Here, we provide insights into the variation in the loading level with the eigenvalues of the reduced-dimensional covariance matrix, and derive the upper and lower boundaries of the diagonal loading level. The specific steps are as follows.

(21) $$h(\hat{\overset{˘}{b}},\mu )=\sum_{n=1}^{N}\frac{\lambda_{n}{\left|c_{n}\right|}^{2}}{{\left(\mu +\lambda_{n}\right)}^{2}}=1$$

Next, we derive an upper bound on μ; due to the inequality $a^{2}+b^{2}\geq 2ab$, we can rewrite (21) as

(22) $$\begin{array}{cc} h(\hat{\overset{⌣}{b}},\mu )= & \sum_{n=1}^{N}\frac{\lambda_{n}{\left|c_{n}\right|}^{2}}{{\left(\mu +\lambda_{n}\right)}^{2}}=1\leq \sum_{n=1}^{N}\frac{\lambda_{n}{\left|c_{n}\right|}^{2}}{4\mu \lambda_{n}}\\ 1\leq & \sum_{n=1}^{N}\frac{{\left|c_{n}\right|}^{2}}{4\mu }=\frac{\sum_{n=1}^{N}{\left|c_{n}\right|}^{2}}{4\mu }\\ \mu \leq & \frac{\sum_{n=1}^{N}{\left|c_{n}\right|}^{2}}{4} \end{array}$$

To avoid a trivial solution, we require that μ>0, $h(\hat{\overset{˘}{b}},\mu )$ is a monotonically decreasing function. The eigenvalue of Equation (18) is replaced with the maximum value $\lambda_{max}$, which gives

(23) $$\begin{array}{cc} \sum_{n=1}^{N}\frac{\lambda_{n}{\left|c_{n}\right|}^{2}}{{\left(\mu +\lambda_{n}\right)}^{2}}= & 1\geq \sum_{n=1}^{N}\frac{\lambda_{\max}{\left|c_{n}\right|}^{2}}{{\left(\mu +\lambda_{max}\right)}^{2}}\\ \mu \geq & \sqrt{\sum_{n=1}^{N}\lambda_{\max}{\left|c_{n}\right|}^{2}}-\lambda_{max} \end{array}$$

Similarly, all the eigenvalues are replaced with the minimum value $\lambda_{min}$, which gives the following upper bound on μ:

(24) $$\mu \leq \sqrt{\sum_{n=1}^{N}\lambda_{min}{\left|c_{n}\right|}^{2}}-\lambda_{min}$$

Thus, the upper and lower boundaries of the diagonal loading level obtained through synthesis are

(25) $$\sqrt{\sum_{n=1}^{N}\lambda_{max}{\left|c_{n}\right|}^{2}}-\lambda_{max}\leq \mu \leq min\left\{\sqrt{\sum_{n=1}^{N}\lambda_{min}{\left|c_{n}\right|}^{2}}-\lambda_{min},\frac{\sum_{n=1}^{N}{\left|c_{n}\right|}^{2}}{4}\right\}$$

On this basis, the IRDRCB adds boundaries, stopping criteria and the bisection method. Once the diagonal loading level found by the Newton search exceeds the upper and lower boundaries, the stopping criteria immediately terminates the iteration process, and then reselects the initial value of the Newton iteration method and uses the bisection method to solve the diagonal loading level, ensuring that the diagonal loading level conforms to the boundaries.

A summary of the steps for computing the diagonal loading level is as follows.

Step (1) Set the upper boundaries $\mu_{max}=min\left\{\sqrt{\sum_{n=1}^{N}\lambda_{min}{\left|c_{n}\right|}^{2}}-\lambda_{min},\frac{\sum_{n=1}^{N}{\left|c_{n}\right|}^{2}}{4}\right\}$, lower boundaries $\mu_{min}=\sqrt{\sum_{n=1}^{N}\lambda_{max}{\left|c_{n}\right|}^{2}}-\lambda_{max}$, initial value $\mu_{1}=eps$, convergence error Err and initial iteration value i=1.

Step (2) Update the Lagrange multiplier $\mu_{i+1}=\mu_{i}+\frac{\sum_{n=1}^{N}\frac{\lambda_{n}{\left|c_{n}\right|}^{2}}{{\left(\mu_{i}+\lambda_{n}\right)}^{2}}-1}{\sum_{n=1}^{N}\frac{2\lambda_{n}{\left|c_{n}\right|}^{2}}{{\left(\mu_{i}+\lambda_{n}\right)}^{2}}}$; let $c=U^{H}\overset{˘}{\bar{b}}$, where $c_{n}$ is the nth element of c.

Step (3) If $\mu_{min}\leq \mu_{i}\leq +\mu_{max}$ is satisfied, update i=i+1 and go to Step (1) until $\sum_{n=1}^{N}\frac{\lambda_{n}{\left|c_{n}\right|}^{2}}{{\left(\mu_{i}+\lambda_{n}\right)}^{2}}-1<Err$ is solved, and the optimal diagonal loading level $\mu_{opt}=\mu_{i}$; otherwise, go to Step (4).

Step (4) Compute the optimal diagonal loading level by using the bisection method; set the initial value $\mu_{1}=\left(\mu_{max}+\mu_{min}\right)/2$.

Step (5) If $\sum_{n=1}^{N}\frac{\lambda_{n}{\left|c_{n}\right|}^{2}}{{\left(\mu_{i}+\lambda_{n}\right)}^{2}}-1<Err$ is satisfied, the optimal diagonal loading level $\mu_{opt}=\mu_{i}$. Otherwise, go to Step (6).

Step (6) If $\sum_{n=1}^{N}\frac{\lambda_{n}{\left|c_{n}\right|}^{2}}{{\left(\mu_{i}+\lambda_{n}\right)}^{2}}-1>0$ is satisfied, update $\mu_{max}=\mu_{i}$; otherwise, update $\mu_{min}=\mu_{i}$.

Step (7) Update i=i+1 and the Lagrange multiplier $\mu_{i}=\left(\mu_{max}+\mu_{min}\right)/2$, and go to Step (5).

## 3. Numerical Examples

### 3.1. Complexity Analysis

Here, the complexities of the IRDRCB method, the RCB method, the Automatically Calculated Diagonal Loading Robust Beamforming (ACDLRB) method and the Eigen-subspace Robust Beamforming (ESRB) method were analyzed. The operation counts of the IRDRCB method and the RCB method are summarized in Table 1 and Table 2. M represents the number of arrays, and N represents a descending dimension. The results from the calculations in each step are summed in Table 1 and Table 2. The IRDRCB method requires $O\left(\frac{4}{3}N^{3}+3N^{2}+N^{2}M+5N+3M+n_{iter}6N\right)$, and the RCB method requires $O\left(\frac{4}{3}M^{3}+2M^{2}+3M+n_{iter}6M\right)$. In addition, the complexity of the ACDLRB method is $O(3M^{3}+M^{2})$, and the complexity of the ESRB method is $O(5M^{3}+M^{2})$.

**Figure 1:**
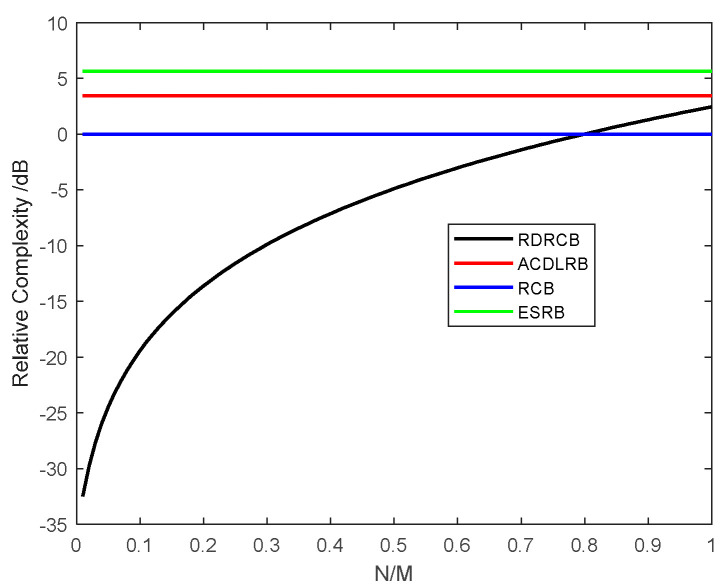
The complexity of RCB, ACDLRCB, ESRB and the IRDRCB with the sensor number fixed at M=100 but with varying rank: N=1~100.

We compare the complexity of RCB, IRDRCB, ACDLRB and ESRB with the sensor number fixed at M=100, but we vary the rank: N=1~100. As can be seen from Figure 1, when the value of τ=N/M is smaller than 0.8, the complexity of the IRDRCB is much better than that of RCB. However, when the value of τ=N/M exceeds 0.8, the IRDRCB requires a larger amount of computation. Figure 1 also shows that the complexity of the IRDRCB is much better than that of ACDLRB and ESRB when τ is less than 1. In summary, the reduced rank N affects computational complexity.

**Figure 2:**
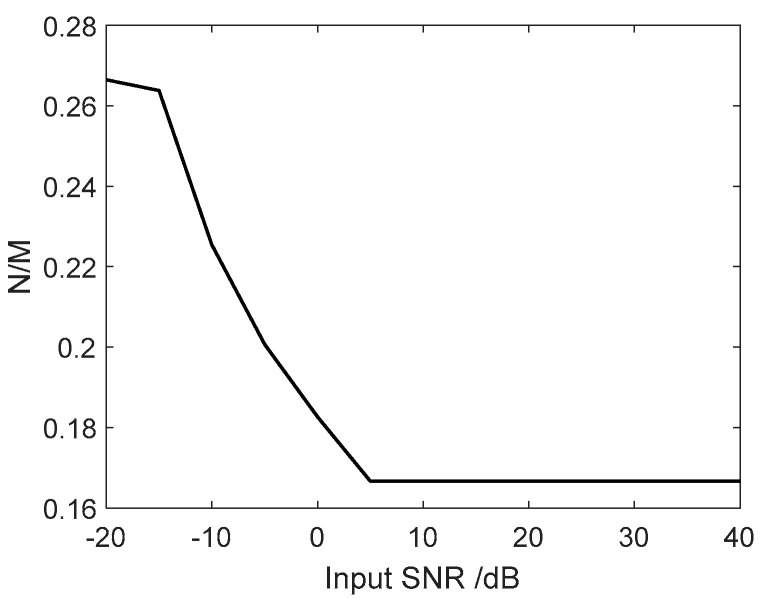
Variation in the relative τ=N/M level with the SNR.

Here, we investigate how the value of τ=N/M varies with the SNR. As we can see from Figure 2, the value of τ=N/M falls within a range of $\left[0.1,\;0.3\right]$. It can be seen that the covariance matrix is reduced when using the Krylov method, and the complexity of the IRDRCB is about 15%~20% that of the RCB method.

### 3.2. Output SINR Versus Input SNR

We now compare the performance of the Capon, RCB and IRDRCB methods when the input signal-to-noise ratio varies. In this example, we assume a linear array with M=10 sensors that are arranged with half-wavelength spacing between adjacent sensors, and we assume that the desired signal, noise and interferers are uncorrelated and stationary.

**Figure 3:**
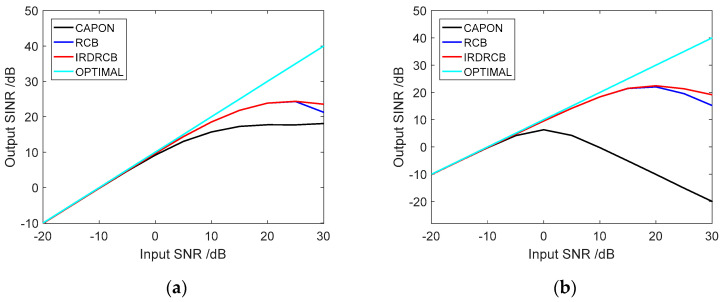
Comparison of the SINR of the Capon, RCB and the IRDRCB with ε=0.5 as the SNR varies: (**a**) SINR with no steering error; (**b**) SINR with a steering error of 0.5°.

We assume that there is a desired signal with an SNR roughly varying from −20 dB to 30 dB from the direction 0°, and that there are two interfering sources with INRs of 30 dB and 30 dB from the directions −20° and 20°, respectively. Figure 3a shows the SINR versus input SNR curves obtained using the Capon, RCB and the IRDRCB with ε=0.5 for no steering vector error. Clearly, the performances of the diagonal loading methods are almost the same at lower SNRs, indicating that the norm constraints are not activated. As the input SNR increases, the SINR curves of all the loading class adaptive algorithms gradually deviate from the optimal output curve, and the performance degradation of the Capon is more obvious than that of the IRDRCB and RCB methods. Moreover, the proposed IRDRCB achieves a performance that is consistently close to, or even better than, that of the RCB methods for all values of SNR. Figure 3b shows the SINR versus input SNR curves obtained using the Capon, RCB and the IRDRCB with ε=0.5 for a steering vector error of 0.5°. Due to the cancelation of the desired signal (the sensor array mismatch between the nominal and actual signal steering vectors), the Capon output SINR first increases and then decreases with an increase in the input SNR. To a certain extent, the IRDRCB and RCB methods address the problem with look direction mismatches and obtain a higher signal-to-noise ratio gain, and when the input signal-to-noise ratio is 20 dB, both the RCB and IRDRCB methods suffer from severe performance degradation regarding SINR gain.

### 3.3. Output SINR Versus the Number of Snapshots

**Figure 4:**
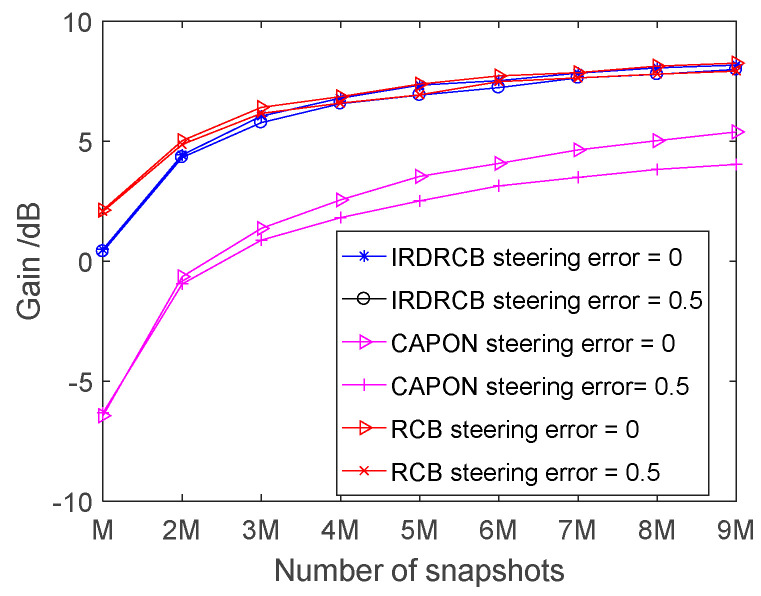
Comparison of the SINR of the Capon, RCB and IRDRCB methods as the number of snapshots varies.

Figure 4 displays the SINR versus the number of snapshots obtained using the Capon, RCB and IRDRCB with ε=0.5. As expected, the larger the number of snapshots, the better the interference suppression capability of all the methods. In contrast, the SINR of RCB is much better than that of both the Capon and the IRDRCB when the number of snapshots is small; for example, when the number of snapshots is M, the SNR gain of RCB is 2 dB higher than that of the IRDRCB and 8 dB higher than that of the Capon. As the number of snapshots increases, RCB and the IRDRCB perform similarly but better than the Capon regardless of the level of steering error. Note that when the number of snapshots is large, there is a large difference in the output SINR of the Capon between the conditions of no steering error and a steering error of 0.5°. However, the level of steering error has a small effect on the variation in the SINR of RCB and the IRDRCB as the input SNR is varied.

### 3.4. Azimuth Spectra

**Figure 5:**
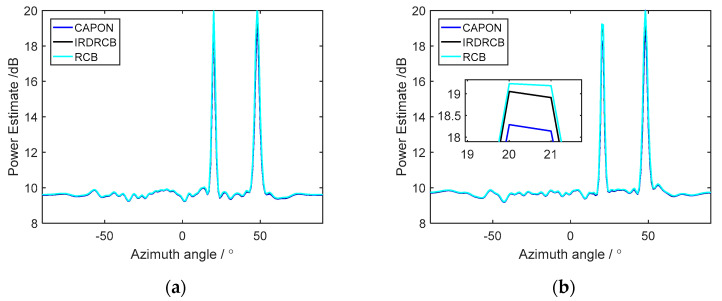
Power estimates versus the steering direction θ obtained using the Capon, RCB and IRDRCB methods: (**a**) power estimate with no steering error; (**b**) power estimate with a steering error of 0.5°.

Let us assume that there are two incident signals with SNRs of 20 dB and 20 dB from the directions 20°and 48°, respectively. Figure 5a shows that both signals have no steering vector error; as can be seen in Figure 5a, the Capon, RCB and IRDRCB methods with ε=0.5 provide excellent power estimates for both signals in this example. Figure 5b shows that there is no steering vector error for the signal from the direction 48°, while the signal from the direction 20° is perturbed with a steering vector error of 0.5°. As can be seen in Figure 5b, the power estimates of RCB and the IRDRCB are almost the same but still substantially underestimate the signal powers from the direction 20°, and the Capon methods suffer from degradation in SOI power estimation accuracy.

### 3.5. Experimental Data Results

**Figure 6:**
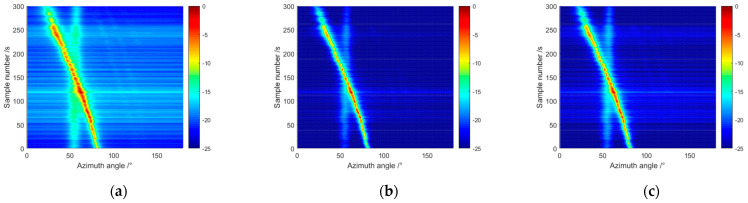
Comparison of the bearing-time records when using the Capon, RCB and IRDRCB methods: (**a**) Capon; (**b**) RCB; (**c**) IRDRCB.

**Figure 7:**
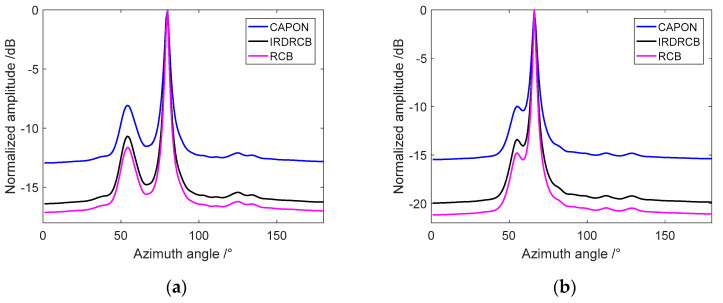
The azimuth spectra for samples at 10 s and 100 s: (**a**) 10 s; (**b**) 100 s.

We proceeded to examine the performance of these methods using experimental data. The beamformed frequency-domain data were inverse Fourier transformed to produce time-series beam data, which were integrated and then magnitude-squared to produce a bearing-time record (BTR). Figure 6a–c show the BTRs obtained by processing 300 s of experimental data for the Capon, RCB and the IRDRCB with ε=0.5, respectively. For the BTR samples, the angle of arrival (AOA) of the interferer is $\theta_{1}=50{}^{\circ}$; the AOA of the target varies from 100° to 30°, where the AOA is measured along the axis of the flank array. Figure 7a,b show the azimuth spectra for samples at 10 s and 100 s, respectively. Clearly, the performance of the IRDRCB is almost indistinguishable from that of RCB; both RCB and the IRDRCB have a larger dynamic range than the Capon, so their background is cleaner and the target track is more prominent. It is clear from the azimuth spectra that the azimuth resolution of RCB and the IRDRCB is similar and better than that of the Capon. By analyzing the azimuth spectra for samples at 10 s, it was found that the SNR gain of the IRDRCB was about 4.5 dB higher than that of the Capon and 1.5 dB lower than that of RCB. Upon analyzing the azimuth spectra for samples at 100 s, it became clear that the IRDRCB provided at least a 3 dB gain over the SDAS for the target compared to the Capon methods. The N/M varied from 5/54 to 7/54 when using the IRDRCB with experimental data. The trial results show that the IRDRCB can obtain a 3~5 dB gain compared with conventional adaptive beamforming, and the computational complexity is reduced by 15% to 20% compared with that of RCB. In summary, the IRDRCB provides excellent performance at a significantly reduced computational complexity and with robustness against array steering vector error and noise.

## 4. Conclusions

Existing RDRCB methods suffer severe performance degradation when the Lagrange multiplier is invalid; an IRDRCB method robust against an invalid diagonal loading level and ASV mismatch was proposed. We provided insights into the variation in the loading level with the eigenvalues of the reduced-dimensional covariance matrix, and derived the upper and lower boundaries of the diagonal loading level. The simulation and experimental data results demonstrate that the IRDRCB provides excellent performance at a significantly reduced computational complexity and with robustness against array steering vector error and noise. Computational complexity is reduced by 15% to 20% compared with that of RCB, and the obtained gain is 3~5 dB compared with conventional adaptive beamforming. The IRDRCB significantly lowers computational complexity compared to standard RCB, making it highly suitable for real-time implementation. The method’s ability to scale efficiently with parallel processing and fast approximate solutions makes it an ideal candidate for real-time sonar and radar systems, where fast, reliable signal processing is crucial. With continued advancements in hardware acceleration, the potential of the IRDRCB in real-time processing will only grow further.

## Figures and Tables

**Table 1:** Online operation counts for RCB.

${\overset{˘}{R}}_{x}=\overset{˘}{U}\overset{˘}{\Lambda }{\overset{˘}{U}}^{H}$	$O\left(\frac{4}{3}M^{3}\right)$
Solve for $\hat{\overset{˘}{a}}$	$O\left(M\left(M+1\right)+n_{iter}6M\right)$
${\hat{a}}_{0}=E^{-\frac{1}{2}}\hat{\overset{˘}{a}}$	$O\left(M\right)$
${\hat{W}}_{RCB}$	$O\left(M\left(M+1\right)\right)$

**Table 2:** Online operation counts for IRDRCB.

Evaluate DRT	$\;O\left(NM\left(M+5\right)\right)$
$R_{y}=\Lambda_{CG}$	$O\left(M\left(M+1\right)\right)$
$\bar{b}=D^{H}\bar{a}$	$O\left(NM\right)$
$\overset{˘}{b}=\Lambda_{CG}^{-\frac{1}{2}}\bar{b}$	$O\left(N\right)$
From M	$O\left(N^{2}\left(M+2\right)\right)$
EVD(M)	$O\left(\frac{4}{3}N^{3}\right)$
$\mathrm{Solve}\;\mathrm{for}\;\hat{\overset{˘}{b}}$	$O\left(N\left(N+1\right)+n_{iter}6N\right)$
${\hat{b}}_{0}=\Lambda_{CG}^{\frac{1}{2}}\hat{\overset{˘}{b}}$	$O\left(N\right)$
${\hat{W}}_{IRDRCB}$	$O\left(2N\right)$

## Data Availability

Data are contained within the article.
